# Why Does Psychotherapy Work and for Whom? Hormonal Answers

**DOI:** 10.3390/biomedicines10061361

**Published:** 2022-06-09

**Authors:** Susanne Fischer, Sigal Zilcha-Mano

**Affiliations:** 1 Institute of Psychology, Clinical Psychology and Psychotherapy, University of Zurich, 8050 Zurich, Switzerland; 2 Department of Psychology, University of Haifa, Haifa 31905, Israel; sigalzil@gmail.com

**Keywords:** alliance, cognitive behavioural therapy, cortisol, oestradiol, oxytocin, psychotherapy

## Abstract

The questions of for whom and why psychotherapy is effective have been the focus of five decades of research. Most of this knowledge is based on self-report measures. Following the biopsychosocial model of mental disorders, this article explores the potential of hormones in answering these questions. The literature on cortisol, oxytocin, and oestradiol in psychotherapy was systematically searched, focusing on (a) baseline hormonal predictors of who may benefit from psychotherapy and (b) hormonal changes as indicators of therapeutic change. The search was limited to depression and anxiety disorders. In sum, the findings show that, of all three hormones, the role of cortisol is most established and that both cortisol and oxytocin are implicated in psychotherapy, although a causal role is still waiting to be demonstrated. Moreover, there is a differential role of hormones in the psychotherapy of depression versus anxiety. The directions of research mapped in this article may elucidate how psychotherapy can be selected to match patients’ endocrine states and how hormonal levels can be manipulated to improve outcomes.

## 1. Introduction

Psychotherapy is a standard first-line treatment for some of the most prevalent mental disorders, including depression and anxiety disorders [1,2,3]. In its early days, psychotherapy research was mainly concerned with the efficacy and effectiveness of various therapies, such as cognitive behavioural therapy (CBT) or psychodynamic psychotherapy. Recently, its focus has shifted towards an improved understanding of the mechanisms underlying successful treatments. This research is often referred to as “what works for whom and why,” and has traditionally been divided into two lines of inquiry: (a) studies on predictors of treatment response (addressing the “for whom”), and (b) studies on indicators of treatment response (addressing the “why”). Pursuing these two lines of inquiry is pivotal as, approximately, only 50% of patients with depression or anxiety disorders show sufficient responses to standard first-line psychotherapy [4,5].

The vast majority of psychotherapy research focusing on the questions of “what works for whom and why” has relied on self-report and clinical interviews, producing a wealth of data on *predictors* and *indicators* of therapeutic change. Common prognostic factors include illness severity or comorbidity with other mental disorders, whereas the most studied process variable is the quality of the therapeutic alliance [6] The accumulated research produced many mixed results and few consistent findings. A potential contributor is the almost exclusive reliance on what individuals are able and interested in reporting when they are answering questionnaires or participating in clinical interviews.

With the advent of the biopsychosocial model [7], mental disorders have begun to be understood as the result of a complex interplay between biological, psychological, and social factors. The last decades have witnessed an increasing interest in biological markers of mental disorders. This trend is also evident in the proposition of the Research Domain Criteria, a framework to study biological signals and processes of mental disorders, along with behaviour [8]. Whereas in the earlier days of biological research, there was mainly a quest for markers of a specific diagnostic status (e.g., the presence of a major depressive disorder), this was followed by the exploration of the prognostic and evaluative potential of biological parameters in relation to treatment outcomes. One of the most fruitful research areas in this regard is psychoneuroendocrinology, a cross-disciplinary field devoted to the interactions between the mind, brain, and endocrine systems [9]. However, as with biomarkers in general, the bulk of research in mental disorders has been conducted in an etiological or diagnostic context, whereas the potential use of hormones in psychotherapy has only recently begun to be recognised.

**Figure 1:**
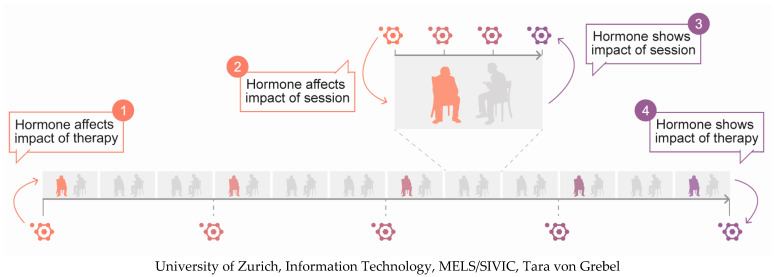
Framework for the investigation of hormones in psychotherapy. Direction: hormones as predictors (1, 2) or indicators (3, 4) of therapeutic changes. Level: treatment (1, 4) or session (2, 3).

The aim of the present review is to provide an up-to-date account of the extent to which hormones are predictive and indicative of psychotherapeutic changes. For this purpose, the end products of three major endocrine systems (i.e., cortisol, oxytocin, and oestradiol) are briefly described with respect to their most important trait- and state-like determinants. This is followed by an outline of their links with cognitive, emotional, and behavioural domains of functioning that are relevant in a therapeutic setting. Finally, the current state of research on how these hormones affect psychotherapy and are affected by it is synthesised. Apart from differentiating between baseline predictor and change indicator studies, two levels of investigation are distinguished: the treatment level and the session level (see Figure 1). Whereas the treatment level provides a global overview of the changes that occur, the session level sheds light on potential mechanisms of action during individual appointments. The review is restricted to depressive and anxiety disorders since, to date, nearly all research on the relationship between hormones and psychotherapy has been dedicated to these conditions.

## 2. Cortisol

The release of the glucocorticoid cortisol from the adrenal cortex is the result of a cascade of actions which take place within the hypothalamic–pituitary–adrenal axis [10]. After the secretion of corticotropin-releasing hormone (CRH) in the paraventricular nucleus of the hypothalamus and its entering of the portal vasculature, adrenocorticotropic hormone (ACTH) is released from the anterior pituitary, which, in turn, is transported via the peripheral bloodstream to initiate the synthesis and secretion of cortisol. Cortisol not only mediates metabolic and immune function in the periphery, but also inhibits the release of CRH and ACTH at the central level by binding to mineralocorticoid and glucocorticoid receptors, hence creating a self-regulatory negative feedback loop.

### 2.1. Main Determinants

Cortisol is influenced by several trait- and state-like characteristics. Regarding trait-like characteristics, both older age and male as opposed to female sex have been associated with higher concentrations [11]. In addition, lifestyle factors, such as physical activity, diet, smoking, and alcohol consumption have repeatedly been linked with altered cortisol [12]. Cortisol concentrations are also found to be changed after extreme and/or prolonged psychosocial stress. Childhood trauma appears to be linked with lower basal cortisol in concert with higher cortisol reactivity [13], whereas the findings on chronic stress point to higher levels at the onset of stress and lower levels thereafter [14].

Regarding state-like characteristics, time of day is a strong determinant of cortisol, with the highest levels observed immediately after awakening and the lowest around 16 h later [15]. In women, this daily rhythm is complemented by a monthly rhythm, with evidence for higher cortisol reactivity around ovulation [12]. Several other behaviours, such as physical exercise, eating, smoking, and drinking have immediate effects on cortisol levels [12]. Finally, uncontrollable social interactions and social-evaluative threats are well-known to elicit a rise in cortisol, with peak levels observed 20 to 30 min after their onset [16]. Interestingly, these effects may extend to longer time periods in the case that individuals ruminate about stressful episodes [17].

### 2.2. Effects on Cognition, Emotion, and/or Behaviour

Given that mineralocorticoid and glucocorticoid receptors are expressed in brain areas such as the hippocampus, amygdala, and prefrontal cortex, it is physiologically plausible that cortisol affects cognitive and emotional functioning. A considerable number of studies have found that cortisol enhances inhibitory functioning while at the same time impairing working memory [18]. An equally substantial amount of research has investigated long-term memory [19]. This line of research has found that cortisol facilitates the consolidation of emotional memories while at the same time impeding their retrieval.

Given these effects, it is not surprising that cortisol has been found altered in chronic states of dysregulated cognitive and/or emotional functioning. Individuals with major depressive disorder are typically characterised by enhanced basal cortisol concentrations when compared to healthy controls [20]. Furthermore, cortisol has been linked to suicidal behaviour, most likely through an impaired ability to make decisions [21]. In individuals with panic disorder, there is fairly consistent evidence in favour of enhanced basal cortisol [22]. Findings in other anxiety disorders are less clear.

### 2.3. Role in Psychotherapy

In order to synthesise the literature on the role of cortisol in psychotherapy, a systematic search in PubMed was conducted until May 2021. The search terms included “cortisol” and “depression” as well as “cortisol” and “anxiety disorder” (including related terms, e.g., “panic disorder”). The studies had to be conducted in a sample of adult patients with any depressive disorder and/or with an anxiety disorder according to the International Classification of Diseases (ICD) [23] or the *Diagnostic and Statistical Manual of Mental Disorders* (DSM) [24] and to include a trial of psychotherapy (mono-treatment). Three systematic reviews/meta-analyses on the role of cortisol in psychotherapy for depression/anxiety, which used these eligibility criteria and were published in 2017 and 2018, respectively, were identified and their results were summarised and complemented by studies published until May 2021. Studies in specific populations (e.g., pregnant women) were excluded. The study results were extracted and reviewed between the two authors.

#### 2.3.1. Cortisol as Predictor of Psychotherapeutic Changes

- Treatment level

A handful of studies have investigated the extent to which cortisol measured before psychotherapy is associated with treatment outcome. In a meta-analysis of patients with depressive disorders, six studies in adults including *N* = 495 patients were identified [25]. The majority of these studies investigated CBT. The meta-analysis suggested that the higher the patients’ pre-treatment cortisol levels, the higher their levels of depression after psychological therapy. In the only study published on the matter since this meta-analysis [26], lower rather than higher levels of cortisol predicted non-responses after psychological therapy. However, this study was conducted in a naturalistic setting, using Improving Access to Psychological Therapies (IAPT) service data, and the majority of patients met diagnostic criteria for both depression and anxiety disorders.

Indeed, in anxiety disorders, the cortisol-treatment outcome pattern appears to differ markedly from to the one observed in depression. Five studies (*N* = 158) conducted in adults with different types of anxiety disorders were identified in a systematic review of the literature [27]. Overall, endogenous cortisol before psychotherapy did not appear to relate to treatment outcomes. In three additional studies on panic disorder published since the systematic review the findings were equivocal, with both negative [28] and mixed [29,30] relationships between cortisol and measures of treatment response reported. In contrast, the systematic review revealed consistent evidence for higher cortisol levels during the initial exposure sessions to predict better therapy outcomes [27]. One study in social anxiety disorder, which was published since the systematic review, reported a somewhat counterintuitive finding, namely that higher cortisol averaged across all exposure sessions predicted reduced improvement in fear [31]. Complementing the line of research on endogenous cortisol, a study in panic disorder and agoraphobia revealed that exposure sessions scheduled at later times during the day, when cortisol is low, were linked with fewer treatment gains than were sessions scheduled at earlier times during the day, when cortisol is high [32]. Furthermore, there is consistent evidence of a greater reduction in phobic fear when patients with specific phobia receive synthetic analogues of cortisol immediately before their initial exposure sessions [33,34,35].

- Session level

No research has been devoted to examining whether endogenous cortisol measured immediately before or during therapy sessions relates to the result of particular interventions or processes. However, one study reported that patients with social anxiety disorder who had received a synthetic analogue of cortisol had greater immediate decreases in fear upon exposure to a phobic stimulus [33].

- Interim summary

Together, these findings suggest a differential role of cortisol in psychotherapy for patients with depressive vs. anxiety disorders. In depression, elevated cortisol before psychotherapy appears to render patients less capable of profiting from treatment. In phobias, low cortisol during initial exposure sessions appears to facilitate patients’ retrieval of fear memories and to impede the formation of extinction memories, ultimately leading to suboptimal treatment outcomes.

#### 2.3.2. Cortisol as Indicator of Psychotherapeutic Changes

- Treatment level

Several studies have investigated the extent to which cortisol changes over the course of psychotherapeutic treatment. In a systematic review of the literature, seven studies in adult patients with depressive disorders (*N* = 273) and eight studies in adult patients with anxiety disorders (*N* = 243) were identified [36]. In studies on depressive disorders, the majority of patients received some form of CBT. Yet, in employing a wide range of different cortisol sampling and analysis protocols, this body of work proved too heterogeneous for meta-analysis. Interestingly, the majority of the included studies, did, however, provide some indication for decreases in cortisol during or in the aftermath of these therapies [37,38,39,40,41,42,43]. In anxiety disorders, all studies used CBT, but otherwise, the literature was also highly heterogeneous, which prevented the authors from undertaking a meta-analysis [36]. Interestingly, the included studies were consistent in showing declines in cortisol over the course of CBT for generalised anxiety disorder [43,44,45,46,47,48,49,50]. The only study published since the systematic review [31] investigated social anxiety disorder and confirmed an earlier null-finding regarding changes of cortisol during CBT [45].

- Session level

Few studies have attempted to determine whether cortisol varies as a consequence of therapeutic interventions. Interestingly, confronting patients with phobic stimuli has generally not been found to elicit a significant cortisol response [27,44,45,48]. A notable exception is a recent study in social anxiety disorder, which observed significant increases in cortisol within exposure sessions; however, these increases appeared to be driven by less than a third of the patients [31]. Interestingly, in a study in which patients with agoraphobia and their therapists were analysed concomitantly, differential trajectories of cortisol were observed across exposure sessions: whereas patients’ cortisol levels were highest 60 min after exposure, the therapists’ cortisol levels were highest shortly before exposure, with steady declines thereafter [51]. In a follow-up study in patients with specific phobia, steady declines in cortisol over the course of the exposure session were found in both patients and therapists [52].

- Interim summary

Collectively, these findings suggest that psychotherapy may improve symptoms of depression and generalised anxiety disorder by lowering cortisol levels. At least in generalised anxiety disorder, this may be the end result of a gradual habituation process that occurs during repeated confrontation with situations that previously caused stress.

## 3. Oxytocin

The neuropeptide oxytocin is produced by magnocellular and parvocellular neurons in the supraoptic nucleus and in the paraventricular nucleus of the hypothalamus [53]. Oxytocin is transported to the posterior pituitary by axons, where it is stored and released into blood vessels. In the periphery, the main effects of oxytocin are fostering uterine contractions during labour and milk ejection during lactation by binding to G protein-coupled oxytocin receptors. In the central nervous system, the same receptors are expressed in areas including the amygdala, hippocampus, nucleus accumbens, and cerebral cortex.

### 3.1. Main Determinants

Oxytocin is influenced by several trait- and state-like characteristics. Its concentrations are comparably elevated in older people and in women [54]. Positive interactions with parental caregivers have been found to be followed by higher oxytocin levels in the long term, whereas negative interactions appear to have an opposite effect [55]. Furthermore, there is evidence that emotionally traumatic experiences are associated with lower oxytocin levels [56]. Regarding state-like characteristics, time of day was repeatedly found to be linked to oxytocin concentrations, with concentrations lowest at around 8 a.m. and highest between 7 and 8 p.m. [54]. In women, this diurnal rhythm is complemented by a monthly rhythm, with rises in oxytocin from the early follicular phase to ovulation, and subsequent decreases into the mid-luteal phase [57]. Finally, intimate behaviours, such as sexual intercourse, nursing a child, and affectionate touching have been linked with increases in oxytocin levels [58].

### 3.2. Effects on Cognition, Emotions, and/or Behaviour

Oxytocin is known to exert numerous effects on social cognition and behaviour through its centrally expressed G protein-coupled receptor. A prominent line of research has found that oxytocin enhances the recognition of basic emotions, in particular of fear [59]. Moreover, oxytocin appears to negatively affect long-term memory performance regarding non-emotional material, and to positively affect memory performance regarding emotional material [60]. Further evidence suggests that oxytocin promotes in-group trust [61] and facilitates the expression of positive emotions [59]. In contrast, research is inconsistent about whether oxytocin is altered in individuals with mental disorders that present with difficulties in social cognition and behaviour. Although there is fairly consistent evidence in favour of a role of oxytocin in social anxiety disorder [62], the evidence for its involvement in depressive disorders is equivocal [63].

### 3.3. Role in Psychotherapy

To summarise the literature on the role of oxytocin in psychotherapy, a systematic search in PubMed was conducted until May 2021. The key words were “oxytocin” and “depression” as well as “oxytocin” and “anxiety disorder” (including related terms, e.g., “panic disorder”). All studies had to use a sample of adult patients with any depressive disorder and/or with an anxiety disorder according to the ICD or DSM and to include a prospective application of psychotherapy (mono-treatment) in order to be included in our review. Studies in specific populations (e.g., pregnant women) were not considered. The study results were extracted and reviewed between the two authors.

#### 3.3.1. Oxytocin as Predictor of Psychotherapeutic Changes

- Treatment level

Scant research has investigated the extent to which endogenous oxytocin measured before psychotherapy are linked to treatment response. Regarding depressive disorders, one study was able to demonstrate that the lower the patients’ oxytocin, the lower their degree of change in depression over the course of a cognitive behavioural analysis system of therapy (CBASP) program [64]. Another study in patients with depressive disorders showed that interpersonal difficulties at baseline were associated with lower oxytocin synchrony between patients and therapists during psychodynamic psychotherapy, which, in turn, predicted a less pronounced reduction in depressive symptoms over the course of treatment [65]. Regarding anxiety disorders, one study in patients with social anxiety disorder tested intranasal oxytocin as an adjunct to exposure therapy [66]. Although the overall level of social anxiety at the end of treatment and at one-month follow-up did not differ between the verum and the placebo group, the former group evaluated their own appearance and speech performance more favourably over the course of the exposure sessions. By contrast, a study in patients with spider phobia found no effects of exogenous oxytocin on symptoms after exposure therapy; on the contrary, the placebo group had better outcomes than the oxytocin group at one-month follow-up [67].

- Session level

No studies have tried to determine whether oxytocin concentrations immediately before or during therapy sessions are correlated with therapeutic processes. One study in patients with depressive disorders demonstrated that intranasal oxytocin enhanced state anxiety, and, in patients with higher levels of depression, decreased non-verbal flight behaviour in a mock psychotherapy session in which patients were asked to disclose personal information [68].

- Interim summary

In sum, these studies suggest differential effects of oxytocin on psychotherapy delivered to patients with depressive vs. anxiety disorders. In depression, lower levels of oxytocin appear to render patients incapable of benefitting from treatment, possibly by affecting their behaviour toward the therapist. In social anxiety disorder, oxytocin augmentation appears to have beneficial effects on maladaptive cognitions, although these seem to be short- rather than long-term.

#### 3.3.2. Oxytocin as Indicator of Psychotherapeutic Changes

Hardly any studies have looked into changes of oxytocin in relation to psychotherapy. One study in patients with major depressive disorder undergoing psychodynamic treatment showed that the higher the extent of within-session conflict and confrontational ruptures with the therapist, the greater the patients’ increase in oxytocin [69]. Another study in patients with major depressive disorder undergoing the same treatment showed that reduced proximity seeking toward the therapist was associated with greater increases in oxytocin [70]. These studies suggest that, at least in depressive disorders, modulation of the patients’ oxytocin levels is one mechanism by which the therapeutic alliance may cause change.

## 4. Oestradiol

The gonadal hormone oestradiol is one of the end products of the hypothalamic-pituitary-gonadal axis [71]. Gonadotropin-releasing hormone (GnRH) is released in the hypothalamus, and upon its entering the portal vasculature, follicle-stimulating hormone (FSH) and luteinizing hormone (LH) are secreted from the anterior pituitary. These hormones, in turn, precipitate the production of oestradiol in the ovaries and testes. Oestradiol mainly mediates sexual function and reproduction. However, it also engages in a negative feedback mechanism, with higher levels inhibiting the release of GnRH, LH, and FSH in the brain by binding to oestrogen alpha, beta, and G protein-coupled receptors. Importantly, in women, a switch to a positive feedback loop occurs shortly before ovulation, with increases in oestradiol levels preceding increases in LH and, to a lesser extent, FSH.

### 4.1. Main Determinants

Oestradiol levels are subject to the influence of several trait- and state-like characteristics. Age and sex are the most significant trait-like determinants of oestradiol: whereas pre-menopausal women exhibit higher average oestradiol levels than men, they show marked declines after menopause, thus approaching the concentrations normally found in men [72]. Men, by contrast, appear to show more steady and less dramatic decreases in oestradiol over the lifespan. Moreover, certain lifestyle behaviours also appear to affect oestradiol concentrations. In women, physical activity has repeatedly been linked with diminished oestradiol levels [73]. Furthermore, the intake of combined oral contraceptives is well-known to lower endogenous oestradiol concentrations to levels usually observed in the early follicular phase [74]. Regarding state-like determinants, oestradiol shows pronounced monthly changes in women, being lowest during the early follicular phase, peaking shortly before ovulation, and showing a second increase during the mid-luteal phase. In women, physical exercise appears to evoke acute increases in oestradiol [75].

### 4.2. Effects on Cognition, Emotions, and/or Behaviour

Given that oestrogen receptors are widely expressed in the hippocampus and in the prefrontal cortex, it is not surprising that there is emerging evidence for oestradiol serving as a modulator of cognitive functioning. Several studies suggest that women perform better in spatial and numeric tasks when oestradiol levels are low, and do better in processing, verbal memory, and verbal fluency tasks when oestradiol levels are high [76,77]. Consistent with these findings, there is evidence for altered oestradiol levels in individuals with cognitive difficulties, such as those suffering from depressive disorders. In men, there is tentative evidence for individuals with depressive disorders to have higher basal oestradiol (and lower testosterone) concentrations than healthy controls [78]. In women, a systematic review of the literature has shown that individuals with premenstrual dysphoric disorders may have lower oestradiol levels in the luteal phase [79].

### 4.3. Role in Psychotherapy

To review the literature on the role of oestradiol in psychotherapy, PubMed was searched systematically until May 2021. The key words used were “oestradiol” and “depression” as well as “oestradiol” and “anxiety disorder” (including related terms, e.g., “panic disorder”). In order to be eligible for inclusion, studies had to use a sample of adult patients with any depressive disorder and/or with an anxiety disorder according to the ICD or DSM and to include a trial of any form of psychotherapy (mono-treatment). Studies in special populations (e.g., pregnant women) were excluded. The study results were extracted and reviewed between the two authors.

#### Oestradiol as Predictor of Psychotherapeutic Changes

- Treatment level

Relatively little research has been conducted on the extent to which endogenous oestradiol before psychotherapy relates to treatment response. One study in female patients with spider phobia found that lower oestradiol levels were linked to more phobic fear and avoidance after exposure therapy [80]. In the same study, it was found that the intake of hormonal contraceptives was associated with worse behavioural outcomes after therapy and at follow-up. This finding was complemented by another study in female patients with spider phobia undergoing exposure therapy, which observed smaller reductions in phobic fear in women on hormonal contraceptives, although significant differences emerged only at follow-up [81]. By contrast, in a third study with the same patient population, endogenous oestradiol levels did not predict treatment response to cognitive therapy [82].

- Session level

Only one study has attempted to determine whether oestradiol is associated with the efficacy of particular psychological interventions. This study found that, in female patients with spider phobia, lower oestradiol and the intake of hormonal contraceptives were linked with slower improvements during an exposure session [80].

- Interim summary

When taken together, these findings suggest that, in women with spider phobia, lower oestradiol levels during exposure may hamper fear extinction, hence leading to suboptimal treatment outcomes.

## 5. Discussion

The aim of this review was to evaluate the potential of different endocrine systems in enhancing our understanding of for whom different forms of psychotherapy work and why. We report three main findings (see Table 1 and Table 2). First, based on the available literature, a case can be made for a role of cortisol in psychotherapy, with considerably fewer studies shedding light on the involvement of oxytocin, and hardly any work published on oestradiol to date. Second, hormones are likely to modulate the psychotherapeutic process, whereas the extent to which they are affected by it remains more unclear. Third, the relevance of different hormones in predicting and indicating changes related to psychotherapy varies considerably between patients with depression and those with anxiety.

The finding that cortisol is the most well-researched hormone is not surprising, given the abundant evidence of elevated concentrations in depression, in particular in patients of the melancholic subtype [20]. The finding of higher cortisol predicting non-response to psychotherapy may be interpreted as interference with patients’ ability to benefit from cognitive restructuring or interpretations by an impaired working memory. Should this hypothesis be confirmed by future research, it may be sensible to use behavioural rather than cognitive interventions in the initial sessions of psychotherapy for depression. Fortunately, on average, psychotherapy appears to be able to reduce cortisol levels in this population. This finding is consistent with the notion of depression as a stress-related disorder [83] and with the fact that most modern psychotherapies help patients to better cope with chronic and/or traumatic stress. Further research may test whether treatments with a specific focus on stress and/or treatments that support patients in renouncing lifestyle behaviours with negative effects on cortisol (e.g., smoking and drinking) may accelerate this decrease. In anxiety disorders, there is less consistent evidence for altered cortisol secretion, with the exception of elevated levels in panic disorder [22]. However, as in depression, psychotherapy seems to lower cortisol in generalised anxiety disorder. It would be interesting to know whether this adaptation occurs during repeated “rehearsing” of situations that were causing chronic worrying before therapy. In phobias, cortisol levels do not appear to be changed as a result of therapy, but findings of cortisol as a mediator of fear extinction [84] have prompted researchers to test whether scheduling exposure sessions to match patients’ peak concentrations of cortisol has the potential of improving treatment outcomes. Further developing this notion, it would be sensible to explore whether behaviours known to cause a significant rise in cortisol (e.g., physical exercise) may be used to render the encounters with phobic stimuli more efficacious.

Oxytocin was studied mainly in patients with depressive disorder and social anxiety disorder. The role of oxytocin in depression is still being debated, with a recent meta-analysis of case–control comparisons yielding a null finding [63]. The finding of lower oxytocin predicting less pronounced change in depressive symptoms may be interpreted as resulting from a lower-quality therapeutic alliance. More specifically, attenuated levels of oxytocin in patients may render them less capable of detecting emotions in their therapist, of placing trust in them, and of expressing positive emotions, thus hampering the formation of a sustainable relationship. Interestingly, exogenous oxytocin levels before a mock therapy session were followed by increases in anxiety, and, in patients with more severe depression, by acute decreases in socially avoidant behaviours. These findings are concordant with the social salience hypothesis of oxytocin, according to which its effects are highly dependent on both the context and the person [85,86,87]. They may imply that at least in a subgroup of patients with depressive disorders, special emphasis should be placed on building a strong therapeutic alliance, starting with the early sessions. It remains to be determined whether oxytocin levels may be permanently altered by psychotherapy, and whether certain types of therapies may be more suited to achieve this goal than others. Initial studies have shown that oxytocin varies as a function of the patient-therapist relationship. This observation echoes the notion of the therapeutic relationship as a vehicle to enable corrective interpersonal experiences [88]. In social anxiety disorder, the available evidence is more uniform in showing a beneficial effect of oxytocin on social cognition. Building on this observation, future inquiries may try and utilise endogenous fluctuations in oxytocin or use behaviours that cause acute increases in oxytocin to optimise treatment outcomes in this population. Interestingly, preliminary findings suggest that oxytocin may hamper treatment success if administered to patients with specific phobias before exposure therapy. Further research into how, exactly, oxytocin interferes with the consolidation and retrieval of emotional memories is thus warranted in this population.

Research into the role of oestradiol has only burgeoned in recent years. Although the role of gonadal hormones in depression is increasingly recognized [78,79,89,90], no research has yet examined their role in psychotherapy. By contrast, the results obtained in patients with specific phobia consistently demonstrate that low-oestradiol milieus may interfere with extinction learning, impeding optimal treatment outcomes [91]. As with cortisol, one implication of this observation is that because certain women (e.g., those on hormonal contraceptives) may be less likely to benefit from some interventions for anxiety, such as exposure, other interventions, such as cognitive restructuring, should be prioritised. Alternatively, the exploitation of natural endocrine rhythms or specific behaviours (e.g., physical exercise) may facilitate therapeutic gains in this population.

**Figure 2:**
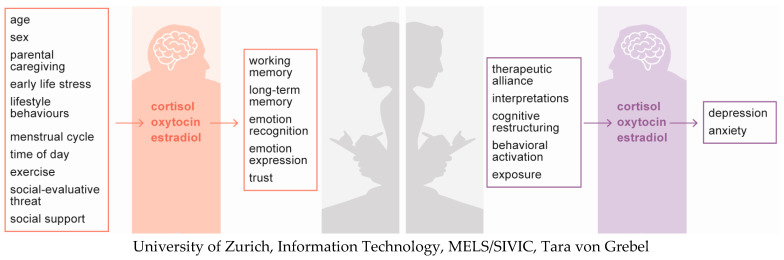
Illustration of how hormones may affect psychotherapy and vice versa. Hormonal determinants and relevant domains of cognitive-behavioural functioning are depicted on the left, whereas relevant therapeutic interventions/processes and outcomes are depicted on the right.

In sum, this article illustrates the potential of hormones in elucidating for whom and why psychotherapy works (see Figure 2). The knowledge that is accumulating from this research may ultimately be used to modify psychotherapy to match patients’ endocrine states and/or to manipulate hormonal levels to achieve better therapy outcomes. However, several potential paths of investigation in this area remain underexplored, and we are therefore far from a complete picture of the role of hormones in psychotherapy.

First, the inter-dependency of predictive and indicative effects of hormones remains under-studied. At the treatment level, only subgroups of patients, such as those with baseline alterations within a specific biological system, may exhibit changes in the very same system in parallel with symptom improvement. Likewise, at the session level, only specific hormonal states may allow for fluctuations in response to the therapeutic process. Second, barely any research has so far taken a dyadic approach to shed light on how hormones and psychotherapy are intertwined. An exception is the study by Levi et al. [92], which has recently shown for the first time that both patient and therapist cortisol concentrations predicted patient post-session affect. Similarly, Zilcha-Mano, Shamay-Tsoory, Dolev-Amit, Zagoory-Sharon, and Feldman [70] have explored for the first time to what extent the patient’s and the therapist’s oxytocin levels were coupled before treatment, and how this evolved as treatment progressed. Furthermore, very little research in this area has studied centrally secreted components of hormonal axes and/or their genetic underpinnings. Related to this, there is a lack of research that has considered interactions between endocrine systems, despite extensive evidence for mutual cross-talk. For instance, hyperactivity within the hypothalamic–pituitary–adrenal axis is often paralleled by hypoactivity within the hypothalamic–pituitary–gonadal axis [93,94]. Luckily, the advent of non-invasive sampling methodologies has paved the way for multi-level assessments of multiple hormonal systems in a way that only minimally interferes with the therapeutic process. The most promising tissues for psychotherapy research to date are saliva [95] and hair [96]. The repeated incorporation of such measures across and within therapy sessions will hopefully allow for a more nuanced answers to the question for whom psychotherapy works and why in the future.

## Figures and Tables

**Table 1:** Summary of studies investigating the relationship between hormones and psychotherapy in individuals with depression.

	Predictor of Outcomes		Indicator of Outcomes	
	Treatment	Session	Treatment	Session
Hormone				
Cortisol	High levels predict worse outcomes [25,26]	-	Levels decrease over treatment [37,38,39,40,41,42,43]	-
Oxytocin	Low levels predict worse outcomes [64] Low synchrony with therapists predicts worse outcomes [65]	Low levels predict lower state anxiety and increased non-verbal flight behaviour during disclosure of personal information [68]	-	Levels increase with conflict and confrontational ruptures [69] Levels increase with reduced proximity seeking [70]
Oestradiol	-	-	-	-

**Table 2:** Summary of studies investigating the relationship between hormones and psychotherapy in individuals with anxiety disorders.

	Hormone as Predictor of Therapeutic Change		Hormone as Indicator of Therapeutic Change	
	Treatment Level	Session Level	Treatment Level	Session Level
Hormone				
Cortisol	Phobias: Low levels during exposure predict worse outcomes [27,31,32,33,34,35]	Social anxiety disorder: Low levels predict greater fear during exposure [33]	Generalised anxiety disorder: Levels decrease over treatment [43,44,45,46,47,48,49,50] Social anxiety disorder: No change [31,45]	Phobias: No change with exposure [27,31,44,45,48]
Oxytocin	Phobias: High levels predict worse outcomes [67] Social anxiety disorder: No effects on outcomes, but high levels predict better evaluation of appearance and speech performance [66]	-	-	-
Oestradiol	Phobias: Low levels predict worse outcomes to exposure therapy but not cognitive therapy [80,81,82]	Phobias: Low levels predict slower improvement during exposure [80]	-	-
